# Lutetium oxodotreotide (^177^Lu-Dotatate) for the treatment of unresectable or metastatic progressive gastroenteropancreatic neuroendocrine tumors: a cost-effectiveness analysis for Scotland

**DOI:** 10.1186/s12885-020-07710-7

**Published:** 2021-01-05

**Authors:** J. Smith-Palmer, O. R. Leeuwenkamp, J. Virk, N. Reed

**Affiliations:** 1 Ossian Health Economics and Communications GmbH, Bäumleingasse 20, 4051 Basel, Switzerland; 2 Advanced Accelerator Applications/A Novartis company, Geneva, Switzerland; 3 Advanced Accelerator Applications/A Novartis company, London, UK; 4 Beatson Oncology Centre, Gartnavel General Hospital, Glasgow, UK

**Keywords:** Cost, Cost-effectiveness, Gastroenteropancreatic neuroendocrine tumors, Radionuclides, Scotland

## Abstract

**Background:**

Gastroenteropancreatic neuroendocrine tumors (GEP-NETs) represent a heterogenous group of tumors. Findings from the phase III NETTER-1 trial showed that treatment of unresectable/metastatic progressive gastrointestinal (GI) NETs with ^177^Lu-Dotatate resulted in a significant improvement in progression-free survival (PFS) and overall survival (OS) compared with best supportive care (BSC) with high dose octreotide long-acting repeatable (LAR) 60 mg. A health economic analysis was performed using input data from clinical studies and data derived from an indirect comparison to determine the cost-effectiveness of ^177^Lu-Dotatate in the treatment of GI-NETs and pancreatic NETs (P-NETs) in Scotland.

**Methods:**

Cost-effectiveness analysis was performed from the payer perspective using a three-state partitioned survival model. In the base case ^177^Lu-Dotatate was compared with BSC in gastrointestinal (GI)-NETs using clinical data from the NETTER-1 trial. A secondary analysis comparing ^177^Lu-Dotatate with BSC, everolimus or sunitinib in patients with P-NETs was also performed using hazard ratios inferred from indirect comparisons. The base case analysis was performed over a 20-year time horizon with an annual discount rate of 3.5% for both costs and clinical outcomes.

**Results:**

For unresectable/metastatic progressive GI-NETs treatment with ^177^Lu-Dotatate led to a gain in quality-adjusted life expectancy of 1.33 quality-adjusted life years (QALYs) compared with BSC due to extended PFS and OS. Mean total lifetime costs were GBP 35,701 higher with ^177^Lu-Dotatate, leading to an incremental cost-effectiveness ratio (ICER) of GBP 26,830 per QALY gained.

In analyses in patients with P-NETs ^177^Lu-Dotatate was associated with ICERs below GBP 30,000 per QALY gained in comparisons with BSC, sunitinib and everolimus.

**Conclusions:**

Cost-effectiveness analyses demonstrated that, in Scotland, from the payer perspective, ^177^Lu-Dotatate at the set acquisition cost is a cost-effective treatment option for patients with unresectable or metastatic progressive GI-NETs or P-NETs.

## Background

Neuroendocrine tumors (NETs) represent a highly heterogenous and diverse class of tumors in terms of histology, clinical presentation and prognosis [1]. The gastrointestinal (GI) tract is the primary site for approximately two thirds of all NETs with other common primary sites including the lungs and the pancreas [1, 2], thus tumors arising from endocrine cells in either the GI tract or pancreas are collectively termed gastroenteropancreatic (GEP) NETs. In the early stages of tumor development GEP-NETs may be asymptomatic, meaning that patients with NETs are frequently diagnosed at an advanced stage, with over 20% of patients presenting with metastatic disease at initial diagnosis [2]. In the UK, recent figures estimate that the annual incidence of NETs is approximately 8 per 100,000 population [3]. More specifically, recent data from the first UK population based study of NETs reported that over the period 2013–2015 the incidence of GEP-NETs was 4.6 per 100,000 population [4]. In Scotland specifically, based on a population estimate of 5.4 million [5], this corresponds to an estimated 432 new cases of NETs per year, of which an estimated 248 will be GEP-NETs, meaning that some individual types of NETs such as GI-NETs meet the criteria for orphan disease status. European prevalence data also scarce, but a recent US-based study reported a prevalence of 0.048% in 2012 [6]. In terms of survival, a recent analysis from the European Neuroendocrine Tumor Society (ENETS) database, which included over 12,000 patients with neuroendocrine tumors across seven European countries, reported median overall survival for all patients of 178 months, although this was influenced by both grade and stage [7].

It is estimated that 80–100% of well differentiated GEP-NETs over-express somatostatin receptors, in particular somatostatin receptor type 2 (SSRT2) on the surface of tumor cells [8]. This property predicts the likely benefit of the use of somatostatin analogues for both diagnostic imaging and targeted tumor treatment. In particular, peptide receptor radionuclide therapy (PRRT) involves somatostatin analogues incorporating a radionuclide, which then delivers targeted cytotoxic radiation directly to the tumor tissue. Specifically, ^177^Lu is a β-emitting radionuclide with a maximum penetration range of 2.2 mm, meaning that it can be directly targeted to somatostatin-receptor expressing tumor tissue with minimal damage to adjacent healthy tissue [9].

In the recent phase III NETTER-1 trial, the efficacy of PRRT using ^177^lutetium oxodeotreotide (hereafter referred to as ^177^Lu-Dotatate [Lutathera]) was assessed in patients with advanced, inoperable, progressive somatostatin-receptor-positive midgut (jejunum, ileum, appendix, or proximal colon) NETs expressing somatostatin receptors [10]. Patients were randomly allocated to either ^177^Lu-Dotatate every 8 weeks (for a total of 4 cycles) plus octreotide 30 mg long-acting release (LAR) given once every 4 weeks or intramuscular octreotide 60 mg alone administered once every 4 weeks. ^177^Lu-Dotatate was administered at a dose of 7.4 GBq per cycle. The primary endpoint was progression-free survival (PFS) and at the time of the primary analysis the median PFS had not been reached in the ^177^Lu-Dotatate arm and was 8.5 months (95% CI: 5.8–9.1 months, *p* < 0.0001, log rank test) in the control octreotide 60 mg arm [10]. In a subsequent *post-hoc* analysis median PFS in the ^177^Lu-Dotatate arm was 28.4 months versus 8.5 months in the octreotide 60 mg arm, resulting in a hazard ratio (95% CI) of 0.21 (0.14–0.33), corresponding to a 79% reduction in the risk for progression or death with ^177^Lu-Dotatate compared with octreotide LAR [11]. Additionally, also in *post-hoc* analyses median OS had not yet been reached in the ^177^Lu-Dotatate arm and was 27.4 months in the octreotide 60 mg arm, resulting in a HR (95% CI) of 0.54 (0.33–0.86) [11]. In terms of safety, findings from the NETTER-1 trial showed that 86% of patients in the ^177^Lu-Dotatate arm experienced adverse events that were considered to be linked to treatment, the most common of which were nausea, vomiting, fatigue or asthenia and diarrhea and 6% of patients in the ^177^Lu-Dotatate arm discontinued treatment owing to adverse events. In the ^177^Lu-Dotatate arm treatment-related adverse events were mostly grade 1 or 2 in severity, the proportion of patients experiencing grade 3 or 4 events was 4% for nausea, 7% for vomiting, 3% for both abdominal pain and diarrhea and 2% for fatigue or asthenia. Additionally, 9, 2 and 1% of patients in the ^177^Lu-Dotatate arm experienced grade 3 or 4 lymphopenia, thrombocytopenia and neutropenia respectively, compared with no patients in the octreotide 60 mg arm [10].

^177^Lu-Dotatate is the first PRRT medicine with marketing authorization for the treatment of GEP-NETs in adults and addresses the unmet clinical need for effective and well-tolerated treatments for NETs [11]. In Scotland and other jurisdictions, Health Service Providers reimbursement of new interventions such as ^177^Lu-Dotatate is contingent on the demonstration of cost-effectiveness relative to the standard of care. In 2008/9 spending on drugs was in excess of GBP 1 billion, representing approximately 10% of total NHS expenditure in Scotland [12]. Data relating to the direct costs of NETs specifically in Scotland are lacking, but a Canadian study noted that following diagnosis the treatment and management costs for patients with NETs were significantly higher than for patients with colon cancer, the most commonly occurring cancer of the GI tract [13, 14]. To evaluate the economic implications of introducing ^177^Lu-Dotatate in Scotland a cost-effectiveness analysis was performed comparing ^177^Lu-Dotatate with adjunctive 30 mg long-acting repeatable [LAR] octreotide with octreotide LAR 60 mg in patients with inoperable, progressive GI-NETs (a secondary analysis was performed to examine the cost-effectiveness of ^177^Lu-Dotatate in patients with inoperable, progressive P-NETs). The results presented in this publication are based on an economic modeling analysis that has been submitted to the Scottish Medicines Consortium (SMC).

## Methods

Two separate cost-effectiveness analyses were performed. In the base case, the cost-effectiveness of ^177^Lu-Dotatate versus octreotide 60 mg (BSC) was performed for patients with unresectable or metastatic, progressive, well differentiated, somatostatin receptor positive GI-NETs. A secondary cost-effectiveness analysis of ^177^Lu-Dotatate was performed for patients with unresectable or metastatic, progressive, well differentiated, somatostatin receptor positive P-NETs in which the comparator treatments were octreotide 60 mg, sunitinib or everolimus. Note that whilst the BSC comparator of octreotide 60 mg is not indicated in P-NETs, the 60 mg dose rather than the dose of 30 mg was used as the BSC comparator for simplicity and consistency in the modeling analysis.

### Model structure

**Fig. 1:**
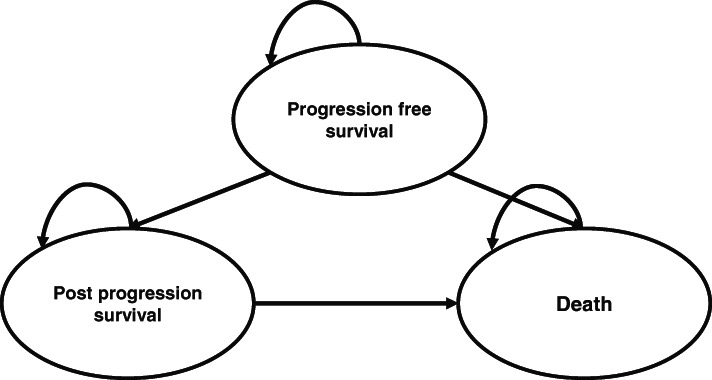
Schematic diagram of partitioned survival model used in the analysis

The analysis was performed using a three-state partitioned survival model [15] constructed in Microsoft Excel (Fig. 1). The three health states were PFS, post-progression survival (PPS) and mortality, and all patients enter the model in the PFS state. State membership is determined based on extrapolated PFS and OS curves. The proportion of patients in the PFS state at any point in time is determined directly from the PFS curve, the proportion of patients in the PPS state is determined by the difference between the OS and PFS curves and the proportion of patients in the “dead” state at any time point is determined by 1 minus the OS curve at that time point. Parametric survival models (Weibull, exponential, Gompertz, lognormal, gamma, logistic) were fitted to time to event data to extrapolate observed PFS and OS over the time horizon of the model. The Weibull model was selected as most appropriate for the base case, based on a combination of visual inspection, Akaike’s Information Criterion and the Bayesian Information Criterion. The cycle length of the model was 4 weeks and half cycle correction was applied.

### Clinical input data: ^177^Lu-Dotatate in GI-NETs

The base case analysis compared ^177^Lu-Dotatate plus octreotide LAR 30 mg with high-dose octreotide LAR 60 mg in patients with inoperable, progressive GI-NET. Clinical input data for the base case analysis were derived from the phase III NETTER-1 trial [10]. Here, median (95% CI) PFS was 28.35 (28.35–not reached) months in the ^177^Lu-Dotatate arm and 8.54 (5.81–11.0) months in the octreotide arm. Median OS was not yet reached in the ^177^Lu-Dotatate arm and 27.37 months for octreotide. It was assumed that patients in the active treatment group received ^177^Lu-Dotatate dose of 7.4 GBq once every 8 weeks for a total of 4 courses. Patients receiving BSC received octreotide LAR 60 mg for symptom control once every 4 weeks. For ^177^Lu-Dotatate, missed doses or dose modifications due to missed doses, toxicity or adverse events were taken into account using relative dose intensity (RDI). The RDI for ^177^Lu-Dotatate was assumed to be 84.4%, based on data from the ERASMUS study. Additionally, as a simplifying assumption it was assumed that upon disease progression all patients switched to octreotide LAR 30 mg once every 4 weeks until death.

### Clinical input data: ^177^Lu-Dotatate in P-NETs

Secondary analyses were performed to compare ^177^Lu-Dotatate with octreotide LAR, sunitinib or everolimus in patients with P-NET using efficacy data based on matched-adjusted indirect comparisons (MAICs) [16]; this method was used to adjust for differences in patient populations between the ERASMUS study (manuscript in preparation) and comparator trials. In the ERASMUS study, analyses were conducted separately for patients with GI-NETs and P-NETs as clinical management practices and available treatments differ between the tumor types. For the P-NET comparisons clinical input data for ^177^Lu-Dotatate-treated patients were sourced from the Dutch cohort of the single-arm phase I/II ERASMUS study (it was decided to limit ERASMUS data to the Dutch cohort only owing to a high proportion of patients with incomplete follow-up data in study centers outside the Netherlands). Efficacy data for sunitinib were sourced from the A6181111 trial [17]. Efficacy data for everolimus were sourced from the RADIANT-3 [18] trial, the comparator in each of which was BSC and equivalence was assumed between BSC and octreotide LAR 60 mg. Hazard ratios for PFS and OS for ^177^Lu-Dotatate versus comparator treatments were derived from MAICs.

Both everolimus and sunitinib were administered orally once daily until progression or discontinuation, everolimus was administered at a dose of 10 mg once daily and sunitinib was administered at a dose of 37.5 mg once daily. For ^177^Lu-Dotatate, everolimus and sunitinib, missed doses or dose modifications due to toxicity or adverse events were accounted for using RDI. For ^177^Lu-Dotatate a RDI of 84.4% was assumed based on findings from the ERASMUS study. For everolimus, the assumed RDI was 79.4%, based on data from the RADIANT-4 trial [19], for sunitinib the corresponding value was 91.3%, which was sourced from the A6181111 [17] trial. As with the base case analysis, as a simplifying assumption it was assumed that upon disease progression all patients switched to octreotide LAR 30 mg once every 4 weeks until death.

### Costs and utilities

Health state utilities utilized in the model were sourced from a UK-based study by Swinburn et al. (2012) in which utility values were elicited using the time trade off (TTO) method (Table 1) [20]. Utility values for adverse events were sourced from published literature (Table 2) [20, 22–24] (only adverse events of grade 3–5 in severity were included in the analysis). Direct costs associated with adverse events were sourced from published literature (Table 2) [25–28].

**Table 1:** Health state utilities used in the base case analysis

Health state	Utility (95% CI)	Reference
PFS	0.771 (0.731–0.810)	20
PPS	0.612 (0.564–0.659)	20
Death	0	―

*I* confidence interval, *PFS* progression-free survival, *PPS* post-progression survival

**Table 2:** Utilities and costs associated with adverse events

Adverse event (grade 3–4)	Utility decrement	Reference	Cost, GBP	Reference
Nausea	0.05	[22]	1.00	Assumed^b^
Vomiting	0.05	[22]	1.00	Assumed^b^
Diarrhea	0.05	[22]	1.00	Assumed^b^
Abdominal pain	0.07	[23]	1.00	Assumed^b^
Thrombocytopenia	0.11	[24]	84.22	[25]
Lymphopenia	0.11	Assumed^a^	1.00	[26]
Leukopenia	0.11	Assumed^a^	160.66	[28]
Stomatitis	0.11	Assumed^a^	385.17	[27]
Fatigue	0.20	[20]	1.00	Assumed^b^
Infections	0.11	Assumed^a^	385.17	Assumed^c^
Asthenia	0.20	Assumed^a^	1.00	Assumed^b^
Anemia	0.12	[20]	375.00	[28]
Pyrexia	0.11	Assumed^a^	32.60	[27]
Hyperglycemia	0.11	Assumed^a^	385.17	Assumed^c^
Neutropenia	0.09	[22]	160.66	[28]
Hypertension	0.11	Assumed^a^	16.34	[27]
Musculoskeletal pain	0.11	Assumed^a^	385.17	Assumed^c^
Flushing	0.11	Assumed^a^	1.00	Assumed^b^
Decreased appetite	0.20	Assumed equal to fatigue	1.00	Assumed^b^

^a^Assumed to be equal to worst disutility in line with NICE TA306 [21]

^b^Assumed, does not have a notable impact on NHS resources at a national level, therefore assumed to be GBP 1.00

^c^No specific data available; assumed to be equal to the highest adverse event cost

Costs were captured from a healthcare payer perspective. Drug acquisition costs (Table 3) and costs associated with drug administration and monitoring as well as adverse event costs were included. Administration and monitoring costs included costs associated with a pharmacist (for treatment preparation), a day ward nurse and outpatient day attendance. Monitoring costs included those associated with computed tomography or magnetic resonance imaging, complete blood counts, blood chemistry and urinalysis tests every 4 weeks and an electrocardiogram every 8 weeks. Treatment acquisition costs were sourced from the British National Formulary [29] and for costs associated with outpatient appointments, laboratory tests, emergency department visits adverse events NHS Reference Costs were used [28]. Costs associated with palliative care were conservatively not included in the base case analysis but a sensitivity analysis including the cost associated with palliative care was performed.

**Table 3:** Unit costs for model treatments

Drug acquisition costs	Dosing and frequency	Unit cost, GBP
^177^Lu-Dotatate 7.4 GBq (200 mCi) (Lutathera)	4 administrations of 7.4 GBq (200 mCi), administered once every 8 weeks	71,500 (for 4 administrations)
Octreotide LAR	60 mg/ 30 mg administered once every 28 days	1 × 30 mg vial, 998.41
Everolimus (Afinitor)	10 mg administered once daily	1 × 30 tab pack (10 mg), 2673
Sunitinib (Sutent)	37.5 mg administered once daily	1 × 30 tab pack (12.5 mg), 784.70

Costs of medications administered prior to or concomitantly with ^177^Lu-Dotatate (i.e. anti-emetics and amino acids) were also included in the analysis with data on doses and frequency of administration sourced from clinical trials

### Sensitivity analyses

A series of sensitivity analyses were performed to determine the key drivers of cost-effectiveness. Several sensitivity analyses were performed around clinical input data, with analyses performed comparing ^177^Lu-Dotatate with octreotide and everolimus using hazard ratios from a mixed treatment comparison. The influence of health state utilities for the PFS state was also examined in analyses utilizing utility values sourced from a UK-based real-world study in GI-NET patients and from the ERASMUS study (data from both studies were mapped to the EQ-5D). The influence of RDI for ^177^Lu-Dotatate was also examined in an analysis that utilized an RDI of 86.4%, sourced from the NETTER-1 trial. In terms of costs, a sensitivity analysis was performed in which costs associated with palliative care were captured. Sensitivity analyses were performed around the discount rate, with an analysis performed using an annual discount rate of 6% for future costs and 1.5% for clinical outcomes (compared with 3.5% per annum for both costs and outcomes used in the base case), sensitivity analyses were also performed using shorter time horizons of 5 and 10 years. Probabilistic sensitivity analysis (PSA) was also performed. Here, 5000 Monte Carlo simulations were performed, variables included and distributions sampled in the PSA were as follows: age (gamma distribution), drug acquisition costs (fixed), ^177^Lu-Dotatate RDI (beta), administration resource use (fixed), administration and monitoring resource use cost (gamma), monitoring resource use frequency (fixed), PFS and PPS utilities (beta), adverse event costs (gamma), and adverse event frequency (gamma or fixed depending on individual event).

### Time horizon, discount rate and perspective

The analysis was performed from the payer perspective, in this instance the Scottish NHS, over a time horizon of 20 years and future costs and clinical outcomes were discounted at a rate of 3.5% per annum in line with recommendations for Scotland [30].

.

## Results

### Cost-effectiveness of ^177^Lu-Dotatate in GI-NET

In the base case analysis, the extended PFS and OS associated with ^177^Lu-Dotatate resulted in an overall incremental gain of 1.33 quality-adjusted life years (QALYs) compared with octreotide LAR 60 mg (3.25 QALYs versus 1.92 QALYs). Notably, the incremental gain in quality-adjusted life expectancy with ^177^Lu-Dotatate in the PFS health state was 1.61 QALYs, reflecting the longer PFS with ^177^Lu-Dotatate relative to octreotide LAR 60 mg (Table 4). Total mean lifetime costs were GBP 35,701 higher with ^177^Lu-Dotatate (GBP 84,990 versus GBP 49,289), primarily driven by higher drug acquisition costs for ^177^Lu-Dotatate in the PFS state. Specifically, 88% of the incremental costs (GBP 41,522) were accrued in the PFS health state, of which GBP 37,263 was attributable to drug acquisition costs. It should also be noted that because PFS and OS were longer for ^177^Lu-Dotatate-treated patients, both treatment and management costs were accrued over a longer period of time. The higher costs combined with improved clinical outcomes resulted in an incremental cost-effectiveness ratio (ICER) of GBP 26,830 per QALY gained for ^177^Lu-Dotatate compared with octreotide LAR 60 mg.

**Table 4:** Summary cost-effectiveness findings in patients with GI-NET

	^177^Lu-Dotatate	BSC (Octreotide LAR)	Δ
Total costs, GBP	84,990	49,289	35,701
Quality-adjusted life expectancy, QALYs	3.25	1.92	1.33
ICER, GBP per QALY gained	26,830		

*BSC* best supportive care, *GI-NET* gastrointestinal neuroendocrine tumor, *ICER* incremental cost-effectiveness ratio, *QALY* quality-adjusted life year

A series of one-way sensitivity analyses were performed to examine key determinants of outcomes (Table 5) and identified changes in time horizon and changes in assumptions relating to efficacy as key drivers of outcomes. In an analysis performed over a time horizon of 5 years the ICER for ^177^Lu-Dotatate versus octreotide 60 mg increased by over 75% relative to the base case to GBP 47,013 per QALY gained; similarly, over a time horizon of 10 years the ICER was GBP 35,986 per QALY gained. In a scenario in which everolimus was the comparator treatment and clinical efficacy data were sourced from an indirect comparison utilizing data from the NETTER-1 trial for ^177^Lu-Dotatate and the RADIANT-4 [19] trial for everolimus ^177^Lu-Dotatate was associated with an incremental gain in quality-adjusted life expectancy of 0.56 QALYs relative to everolimus but lifetime costs were GBP 21,683 higher, resulting in an ICER of GBP 39,169 per QALY gained. Changes in the source data for PFS utility values and the use of the RDI from the NETTER-1 trial had only a minor influence on cost-effectiveness.

**Table 5:** Summary findings of sensitivity analyses for ^177^Lu-Dotatate versus octreotide LAR 60 mg in GI-NET

Analysis	Costs, GBP			Quality-adjusted life expectancy, QALYs			ICER, GBP per QALY	% change from base case
	^177^Lu-Dotatate	Octreotide 60 mg	Delta	^177^Lu-Dotatate	Octreotide 60 mg	Delta		
Base case analysis	84,990	49,289	35,701	3.25	1.92	1.33	26,830	―
Time horizon 5 years	75,715	44,896	30,818	2.37	1.71	0.66	47,013	75.23
Time horizon 10 years	86,710	48,758	37,951	2.95	1.89	1.06	35,986	34.13
Discount rate 1.5% clinical outcomes, 6% costs	80,896	46,711	34,185	3.48	2.00	1.48	23,104	−13.88
Efficacy; using MTC data for ^177^Lu-Dotatate versus octreotide	89,728	49,022	40,706	3.09	1.90	1.19	34,478	28.51
Efficacy; using MTC data for ^177^Lu-Dotatate versus everolimus (RADIANT-4)	89,728	68,045^a^	21,683	3.09	2.53^a^	0.56	39,169	45.99
PFS utility from NETTER-1	84,990	49,289	35,701	3.14	1.90	1.24	28,750	7.16
PFS utility from real world study^a^	84,990	49,289	35,701	3.26	1.94	1.32	26,855	0.09
^177^Lu-Dotatate RDI 86.4% (NETTER-1)	86,370	49,289	37,081	3.25	1.92	1.33	27,866	3.86
Including palliative care costs	91,354	63,464	27,890	3.16	1.73	1.43	19,544	−27.15

^a^Everolimus

^b^Data from analysis of registry data from Guy’s and St Thomas’ Hospital, London. AAA data on file

*HR* hazard ratio, *ICER* incremental cost-effectiveness ratio, *MTC* mixed treatment comparison, *PFS* progression-free survival, *QALY* quality-adjusted life year, *RDI* relative dose intensity

A PSA was also performed, here the ICER was GBP 24,417 per QALY gained. Results of the PSA were also used to construct a cost-effectiveness acceptability curve, analysis of which showed that a willingness-to-pay threshold of GBP 30,000 per QALY gained the likelihood of ^177^Lu-Dotatate being considered cost-effective relative to octreotide 60 mg in GI-NET was estimated at approximately 55%.

### Cost-effectiveness of ^177^Lu-Dotatate in P-NET

Two separate analyses were performed to compare the cost-effectiveness of ^177^Lu-Dotatate with octreotide 60 mg based on clinical input data from the placebo (BSC)-treated arms of the A6181111 [17] and RADIANT-3 [18] trials respectively (Table 6). In the analysis based on the A6181111 trial, treatment with ^177^Lu-Dotatate resulted in an incremental gain in quality-adjusted life expectancy of 2.61 QALYs, whilst the corresponding figure for the analysis based on data from the RADIANT-3 trial was 1.68 QALYs. In both analyses lifetime costs were higher in the ^177^Lu-Dotatate arm, driven by higher drug acquisition costs, which resulted in ICERs of GBP 25,068 and GBP 29,964 per QALY gained based on A6181111 and RADIANT-3 trial data, respectively. It should be noted that although clinical input data for the P-NET analyses were sourced from an MAIC, which adjusts for differences between patient baseline characteristics, the analysis is nevertheless based on an indirect comparison and results should therefore be interpreted with caution owing to between-trial differences.

**Table 6:** Summary cost-effectiveness findings in patients with P-NET

Analysis	^177^Lu-Dotatate	Comparator	Δ
**P-NET, versus octreotide LAR 60 mg**^**a**^			
Total costs, GBP	117,922	52,470	65,452
Quality-adjusted life years, QALYs	4.78	2.16	2.61
ICER, GBP per QALY gained		25,068	
**P-NET versus sunitinib**			
Total costs, GBP	113,423	81,303	32,119
Quality-adjusted life expectancy, QALYs	4.88	2.92	1.96
ICER, GBP per QALY gained		16,390	
**P-NET versus everolimus**			
Total costs, GBP	108,445	70,974	37,472
Quality-adjusted life expectancy, QALYs	4.12	2.69	1.44
ICER, GBP per QALY gained		26,103	

^a^Based on MAIC data obtained from the placebo arm of the A6181111 trial of sunitinib [17]

*GI-NET* gastrointestinal neuroendocrine tumor, *ICER* incremental cost-effectiveness ratio, *LAR* long-acting release, *MAIC* matched adjusted indirect comparison, *P-NET* pancreatic neuroendocrine tumor, *QALY* quality-adjusted life year

Numerical discrepancies due to rounding

When compared with sunitinib in P-NET patients, treatment with ^177^Lu-Dotatate was associated with a projected gain in quality-adjusted life expectancy of 1.96 QALYs (4.88 QALYs with ^177^Lu-Dotatate versus 2.92 QALYs with sunitinib). 75% (1.46 QALYs) of the incremental benefit in quality-adjusted life expectancy was accrued in the PFS state, with the remaining 25% (0.50 QALYs) accrued in the PPS state. Total mean per patient costs were GBP 32,119 higher in the ^177^Lu-Dotatate arm than in the sunitinib arm, which was largely driven by higher drug acquisition and administration costs in the ^177^Lu-Dotatate arm. The gain in quality-adjusted life expectancy combined with higher lifetime costs resulted in an ICER of GBP 16,390 per QALY gained for ^177^Lu-Dotatate arm versus sunitinib.

In the comparison with everolimus, treatment with ^177^Lu-Dotatate was associated a gain in quality-adjusted life expectancy of 1.44 QALYs, 76% of which (1.09 QALYs) was due to increased time spent in the PFS state. Total mean lifetime costs were also GBP 37,472 higher lifetime costs leading to an ICER of GBP 26,103 per QALY gained for ^177^Lu-Dotatate compared with everolimus.

## Discussion

The analysis presented here is one of the first economic analyses to date that examines the cost-effectiveness of treatment with ^177^Lu-Dotatate in patients with unresectable or metastatic GI-NET and P-NET with disease progression and is the first economic analysis of PRRT specific to Scotland. In the analysis in patients with GI-NET, ^177^Lu-Dotatate was compared with octreotide 60 mg LAR. In patients with P-NET ^177^Lu-Dotatate was compared with octreotide 60 mg LAR (although not indicated octreotide 60 mg LAR was used for consistency with the GI-NET analysis and equivalence with BSC was assumed), sunitinib and everolimus, respectively. In all comparisons performed in both GI-NET and P-NET, treatment with ^177^Lu-Dotatate was associated with a projected ICER below GBP 30,000 per QALY gained relative to the comparator treatment. Although no formal willingness-to-pay threshold exists in Scotland, interventions with ICERs below GBP 30,000 are generally regarded as being cost-effective. Consequently, from the healthcare payer perspective, ^177^Lu-Dotatate may be regarded a cost-effective option versus currently available treatments for patients with unresectable or metastatic GEP-NETs experiencing disease progression. Indeed, in July 2018, the SMC published guidance accepting ^177^Lu-Dotatate for use in unresectable or metastatic, progressive, well differentiated, somatostatin receptor-positive GEP NETs in adults on the basis of consideration of clinical evidence from the NETTER-1 and ERASMUS studies and cost-effectiveness analyses performed using the model presented here [11].

GEP-NETs are a heterogenous group of tumors and there are frequently distinct differences in the aggressiveness and genomic and clinical profiles of tumors based on their site of origin [31]. It is possible that the notable difference in the gain in quality-adjusted life expectancy between GI-NET and P-NET patients treated with ^177^Lu-Dotatate when compared with octreotide may be reflective of underlying differences in molecular biology between GI-NET and P-NET. Indeed, response to PRRT has been shown to be influenced by several factors including site of tumor origin and degree of SSRT expression [32]. Despite similar ICERs in comparisons with octreotide, the mean incremental gain in quality-adjusted life expectancy associated with ^177^Lu-Dotatate versus octreotide was 1.33 QALYs for GI-NET, but over 2.6 QALYs for P-NET. Similarly, gains in quality-adjusted life expectancy for P-NET patients treated with ^177^Lu-Dotatate relative to sunitinib and everolimus were 1.96 QALYs and 1.44 QALYs, respectively. Collectively, these results suggest that the incremental clinical benefit associated with the use of ^177^Lu-Dotatate is particularly pronounced in patients with P-NET and that ^177^Lu-Dotatate represents an important advance in the treatment of P-NET, particularly given that historically P-NETs have been associated with poor prognosis. Indeed, the extended PFS and overall survival associated with ^177^Lu-Dotatate have been recognized and this is reflected in guidelines specific to Scotland and also in European guidelines [33, 34]. Specifically, guidance published by the Scottish Neuroendocrine Tumor group advocates the use of radiolabeled somatostatin analogues for patients who have “significant disease demonstrated on ^111^In-octreotide scintigraphy and acceptable renal function.” [34] The clinical evidence base for the Scottish and European guidelines included the phase III NETTER-1 trial and the phase I/II ERASMUS trial, both of which were used to inform the cost-effectiveness analysis presented here. It should be noted that the findings of analysis of ^177^Lu-Dotatate versus sunitinib and everolimus in patients with P-NETs should be interpreted with caution as these are based on indirect comparisons and between-trial differences should be taken into account. At present, the use of indirect comparisons is necessary as there are no data available from head-to-head clinical trials. However, the COMPETE trial [35] (NCT03049189) is an ongoing trial comparing PRRT (^177^Lu-edotreotide) with everolimus in inoperable, progressive, somatostatin-receptor positive GEP-NETs and will provide data that can be utilized in future health economic analyses.

Although the ICERs reported in this analysis were below GBP 30,000 per QALY gained for all comparisons, drugs for orphan indications frequently have higher ICERs relative to more common conditions. For example, in England and Wales, the average ICER for oncology drugs funded by NHS England’s Cancer Drugs Fund (CDF) has been estimated at GBP 75,086 per QALY gained [36]. In the National Institute of Health and Care Excellence (NICE) evaluation of ^177^Lu-Dotatate, NICE concluded that for the GI-NETs ICERs for ^177^Lu-Dotatate versus comparator treatments were within acceptable limits [37]. NICE also deemed that for P-NETs ^177^Lu-Dotatate fulfilled the criteria for end of life care and was considered to be cost-effective versus currently available treatments.

In Scotland, a series of reforms were instigated in 2014, which were designed to increase patient access to new drugs for orphan diseases, such as some types of GEP-NETs, as well as drugs used for end of life care (defined as a disease stage where death usually occurs within 3 years) [38]. The SMC have also previously reported that higher ICERs and/or greater uncertainty may be accepted for new treatments for orphan diseases or for agents that confer a considerable improvement in life expectancy (i.e. a median gain in life expectancy of 3 months or more) or in quality-adjusted life expectancy [39]. The clinical evidence base for ^177^Lu-Dotatate suggest that ^177^Lu-Dotatate does indeed fulfil these criteria in terms of providing substantial clinical benefits relative to the currently available standard of care. In the NETTER-1 trial, median OS in the ^177^Lu-Dotatate was not yet reached at the time of the primary analysis, however, in post-hoc analysis the median PFS was 28.4 months for ^177^Lu-Dotatate and 8.5 months for octreotide, thereby showing that ^177^Lu-Dotatate results in a significant improvement in PFS. The gain in quality-adjusted life expectancy with ^177^Lu-Dotatate was also substantial, conferring an incremental benefit of over 1.3 QALYs relative to all comparators included in the analysis.

Although the survival related benefits are marked, the limitations of the present analysis should be noted to properly contextualize the findings. Firstly, costs associated with palliative care were not included in the analysis, which given the notable differences between different treatments in terms of PFS and OS may have influenced the results. For the secondary analyses, in the absence of head-to-head trial data, it was necessary to utilize clinical input data from indirect comparisons and a single-arm non-randomized study of ^177^Lu-Dotatate. Heterogeneity between different study populations is a key limitation in terms of indirect comparisons; however, for the analyses in P-NET patients MAICs were used to adjust for differences in patient characteristics across studies. A further limitation associated with the use of clinical input data from the A6181111 and RADIANT-3 trials, as noted in a previous analysis performed by Mujica-Mota et al. [40], was the high rate of treatment switching reported in both of these trials. This may in turn have had a considerable impact in terms of the uncertainty around efficacy estimates for sunitinib and everolimus. A further limitation associated with the use of input data from any randomized controlled trial is the generalizability of the efficacy results of trials to routine clinical practice. A proportion of patients encountered in routine clinical practice may have poor prognostic factors such as poor performance status or active brain metastases that would have precluded entry to clinical trials. However, whilst the proportion of patients with poor prognostic factors may influence the effectiveness of ^177^Lu-Dotatate in routine clinical practice, it is also feasible that this would influence the effectiveness of comparator treatments to approximately the same degree and is therefore unlikely to influence the overall conclusions of the cost-effectiveness analysis.

## Conclusions

Overall, the findings of the analysis suggest that, in Scotland, ^177^Lu-Dotatate is associated with substantial survival benefits in patients with GEP-NETs relative to octreotide-containing BSC, sunitinib and everolimus, and is likely to be cost-effective compared with currently available treatments including octreotide, everolimus and sunitinib.

## Data Availability

Input data are available upon reasonable request from the authors.

## Abbreviations

BSC: Best supportive care
GBP: Great British pounds
GEP: Gastroenteropancreatic
GEP-NETs: Gastroenteropancreatic neuroendocrine tumors
GI: Gastrointestinal
ICER: Incremental cost-effectiveness ratio
LAR: Long-acting repeatable
MAICs: Match-adjusted indirect comparisons
NETs: Neuroendocrine tumors
NICE: National Institute of Health and Care Excellence
OS: Overall survival
PFS: Progression-free survival
P-NETs: Pancreatic neuroendocrine tumors
PPS: Post-progression survival
PRRT: Peptide receptor radionuclide therapy
PSA: Probabilistic sensitivity analysis
QALY: Quality-adjusted life years
RDI: Relative dose intensity
SMC: Scottish Medicine Consortium
SSRT2: Somatostatin receptor type 2
TTO: Time trade off
^177^Lu-Dotatate: ^177^lutetium oxodeotreotide
